# Silicone-Acyclovir Controlled Release Devices Suppress Primary Herpes Simplex Virus-2 and Varicella Zoster Virus Infections *In Vitro*

**DOI:** 10.1155/2013/915159

**Published:** 2013-08-04

**Authors:** Carol L. Berkower, Nicole M. Johnson, Stephen B. Longdo, Shenika O. McGusty-Robinson, Samantha L. Semenkow, Barry J. Margulies

**Affiliations:** ^1^Towson University Herpes Virus Lab, Department of Biological Sciences, Towson University, Towson, MD 21252, USA; ^2^McDaniel College, Department of Biology, Westminster, MD 21157, USA; ^3^Georgetown and George Mason University, Advanced Biomedical Science Graduate Certificate Program, Manassas, VA 20110, USA; ^4^Department of Pathology, Johns Hopkins University School of Medicine, Baltimore, MD 21205, USA; ^5^Molecular Biology, Biochemistry, and Bioinformatics Program, Towson University, Towson, MD 21252, USA; ^6^Department of Pharmacology and Molecular Sciences, The Johns Hopkins University School of Medicine, Baltimore, MD 21205, USA

## Abstract

Following initial infection, herpesviruses retreat into a permanent latent state with periodic reactivation resulting in an enhanced likelihood of transmission and clinical disease. The nucleoside analogue acyclovir reduces clinical symptoms of the three human alpha herpesviruses, HSV-1, HSV-2, and VZV. Long-term administration of acyclovir (ACV) can reduce the frequency and severity of reactivation, but its low bioavailability and short half-life require a daily drug regimen. Our lab is working to develop a subcutaneous delivery system to provide long-lasting, sustained release of ACV. Previously, we demonstrated that an implantable silicone (MED-4050) device, impregnated with ACV protected against HSV-1 both *in vitro* and *in vivo*. Here, we extend our *in vitro* observations to include protection against both HSV-2 and VZV. We also demonstrate protection against HSV-2 *in vitro* using MED-4750, a silicone polymer designed for long-term use in humans. When release of ACV from MED-4750 is quantitated on a daily basis, an initial burst of 5 days is observed, followed by a long period of slow release with near-zero-order kinetics, with an average daily release of 1.3923 ± 0.5908 **μ**g ACV over days 20–60. Development of a slow-release implant has the potential to significantly impact the treatment of human alpha herpesvirus infections.

## 1. Introduction

Herpesviruses are among the most prevalent human pathogens, and most individuals are infected with multiple species by the time they reach adulthood. Following primary infection of the human host, herpesviruses retreat into a latent infectious state that persists for the host's lifetime. Periodic activation of the latent infection is associated with an enhanced risk of transmission and may be symptomatic, resulting in characteristic lesions, or asymptomatic [1–5].

The human alpha herpesvirus subfamily includes three members: herpes simplex virus-1 (HSV-1), herpes simplex virus-2 (HSV-2), and varicella zoster virus (VZV). All three establish a primary infection in epithelial cells followed by latent infection of neurons. Among residents of the United States of ages 14–49, seroprevalence of HSV-1 is estimated at 57.7% and HSV-2 at 16.2%, while VZV infection was nearly universal prior to introduction of a childhood vaccine in 1995 [6–9]. Herpesviruses can be reactivated by a variety of physical or emotional stressors. Clinical reactivation of alpha herpesviruses is estimated to occur in roughly 10–30% of infected human hosts, although subclinical shedding occurs with greater frequency [8, 10–12]. Herpesvirus outbreaks in immunocompromised patients are more frequent and harder to control than those in otherwise healthy individuals and can be life-threatening [8, 13–15].

Since the 1980s, the guanosine analogue acyclovir (ACV) and its derivatives, including valacyclovir (VACV; Valtrex, GlaxoSmithKline), have been the first-line drugs for treatment of HSV infection and reactivation [4, 14, 16, 17]. While ACV was a breakthrough in the treatment of herpes, it has several shortcomings. ACV has poor oral bioavailability (~20% of ingested drug is absorbed) and has an elimination half-life in the bloodstream of approximately 3 hours [18–20]. As a result, in order to maintain a constant level of drug sufficient for suppression of clinical disease, pills must be taken on a daily basis, requiring a high level of patient compliance.

Patient compliance has been a particular concern for those attempting long-term prophylactic therapy for genital herpes [21, 22]. This is unfortunate because ACV can suppress reactivation of HSV-2 when it is taken prophylactically over an extended period, reducing both clinical episodes and subclinical shedding and decreasing the likelihood of transmission, with very low toxicity [23–28]. Long-term prophylaxis is also being considered in populations with high rates of HIV transmission, as infection with HSV-2 increases the likelihood of acquiring HIV two- to threefold [29–31].

Reactivation of VZV causes herpes zoster, also known as shingles, which can result in debilitating skin sensitivity [32, 33]. A live attenuated VZV vaccine has been available in the United States since 1995, resulting in dramatic declines in the incidence of chicken pox among children as well as unvaccinated adults, the latter likely due to herd immunity [8]. While efficacy of the VZV vaccine is high, the millions of adults who were exposed to chicken pox as children remain susceptible to reactivation of VZV in the form of shingles and its frequent complication, postherpetic neuralgia (PHN), which can develop into a lasting persistent pain syndrome. Prophylactic ACV can prevent the occurrence of both herpes zoster and PHN [34–36].

Despite the safety and efficacy of long-term ACV use, the challenge of maintaining patient compliance over an extended period limits its utility. For this reason, our lab has been developing a controlled release delivery system using implantable silicone rods impregnated with ACV. These rods are designed to provide long-term delivery of drug at a constant dose, while eliminating the need for patient compliance with a daily drug regimen.

Previously, our lab synthesized a silicone-ACV subcutaneous implant that released ACV with near-zero-order kinetics and protected against HSV-1 infection in cell culture and against HSV-1 reactivation in mice [37]. In this paper, we report on the efficacy of the implant against the two other human alpha herpesviruses, HSV-2 and VZV, in cell culture. We compare release kinetics and *in vitro *efficacy using two different silicone polymers, one of which is approved for long-term implantation in humans, and we compare the efficacy of implants of varying drug : polymer ratios, allowing us to determine the minimum effective dose *in vitro*.

## 2. Materials and Methods

### 2.1. Implant Production

**Figure 1:**
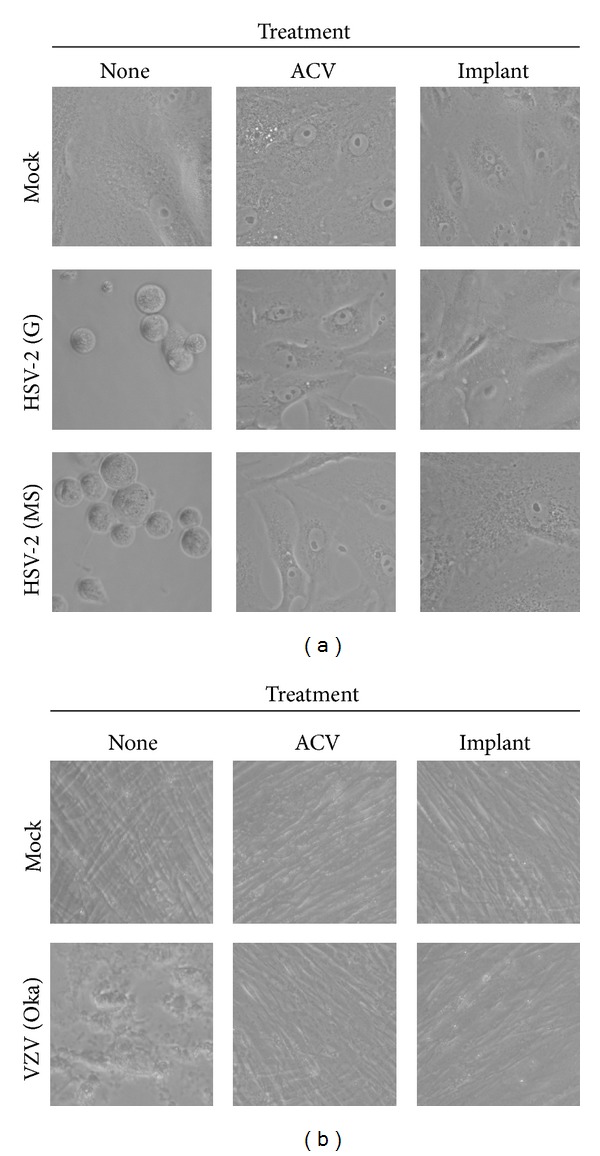
*In vitro *antiviral activity of silicone implants impregnated with ACV against HSV-2 and VZV. (a) Vero cells were grown to near-confluence and received either medium alone (“None”), acyclovir (0.22 *μ*g/mL), or an acyclovir-containing silicone implant (MED-4050). One day later, wells were inoculated with either complete medium alone (“Mock”), 95 pfu of HSV-2 (G), or 95 pfu of HSV-2 (MS). Digital photos were taken 3 days after infection. This experiment was performed six times; representative wells are shown. Each implant was prepared by mixing polymer component A, polymer component B, and ACV in a 1 : 1 : 1 ratio as described in Section 2. (b) MRC-5 cells were grown to near-confluence and received either medium alone (“None”), acyclovir (10 *μ*g/mL), or an acyclovir-containing silicone implant (MED-4050). Two days later, wells were inoculated with either buffer alone (“Mock”) or 150 pfu of VZV. Digital photos were taken 10 days after infection. This experiment was performed three times; representative wells are shown. Each implant was prepared by mixing polymer component A, polymer component B, and ACV in a 1 : 1 : 1 ratio as described in Section 2.

**Figure 2:**
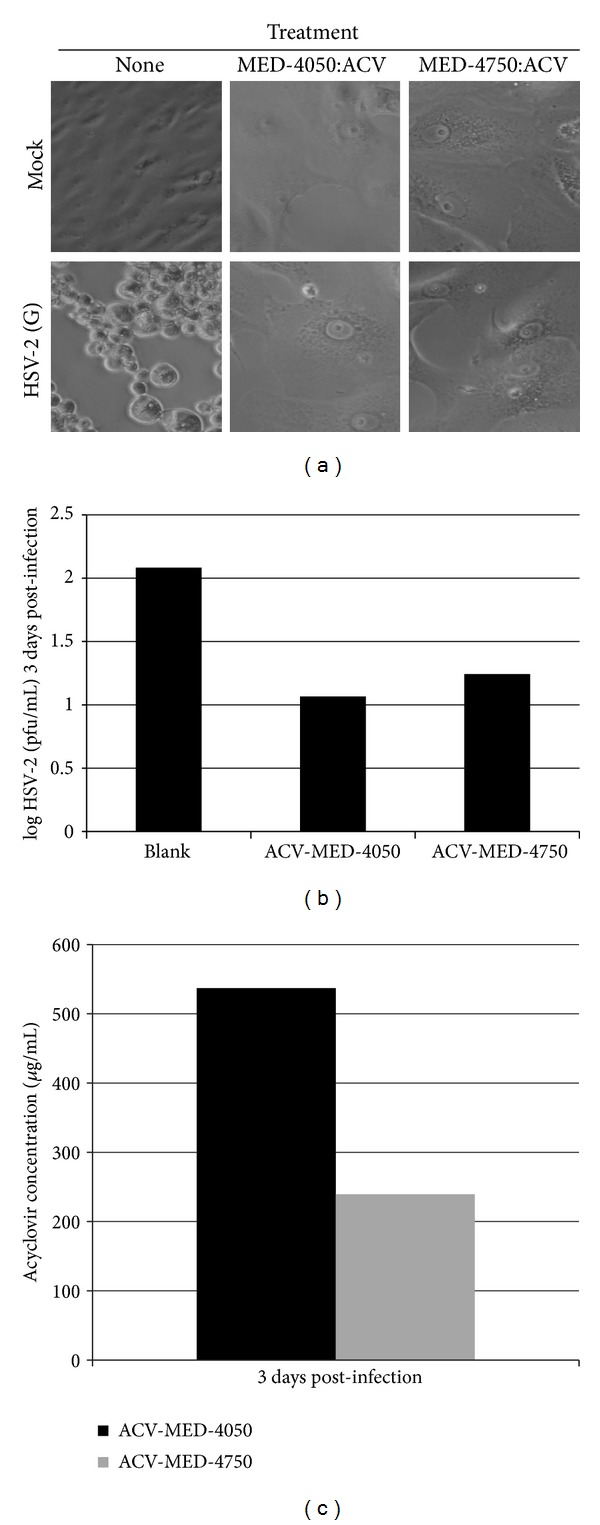
Comparison of *in vitro *antiviral activity of implants made from two different silicone polymers impregnated with ACV. Vero cells were grown to near-confluence in 24-well culture plates. One implant was placed in each well. Two days later, either medium alone (“Mock”) or HSV-2 (G) (95 pfu) was added to the indicated wells. (a) Digital photographs were taken 3 days after infection. Wells in the first column (“None”) received neither implant nor ACV. The remaining wells received MED-4050 or MED-4750 impregnated with ACV, as indicated. Each implant was prepared by mixing polymer component A, polymer component B, and ACV in a 1 : 1 : 1 ratio as described in Section 2. Supernatants (200 *μ*L) were removed from the culture 3 days after infection. (b) A portion of each captured supernatant (100 *μ*L) was used to determine HSV-2 titer. (c) ACV was quantified from the other portion (100 *μ*L) of the recovered supernatant.

**Figure 3:**
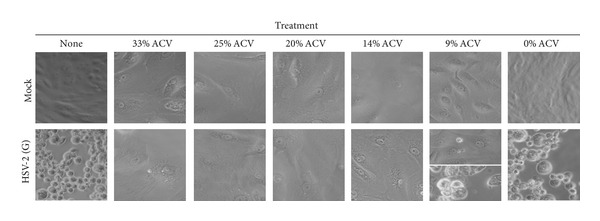
Comparison of antiviral activity of MED-4750 polymers impregnated with varying quantities of ACV. Vero cells were grown to near-confluence in 24-well culture plates. Implants were prepared by mixing MED-4750 component A, MED-4750 component B, and ACV in varying ratios, and one implant was placed in each well. Two days later, either medium alone (“Mock”) or HSV-2 (G) (95 pfu) was added to the indicated wells. Wells marked “None” received no implant; the rest received implant formulated with the indicated percentage of acyclovir. Wells were photographed 3 days after infection. In the split panel of HSV-2 (G)-infected cells incubated with an implant containing 9% ACV; the top and bottom panels were photographed 3 and 5 days after infection, respectively.

**Figure 4:**
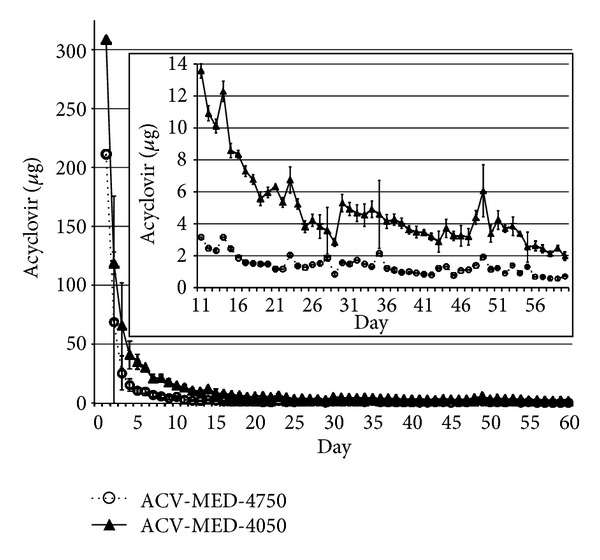
Comparison of *in vitro* release of acyclovir (ACV) from implants made from MED-4050 and MED-4750 polymers. Silicone rods generated from either MED-4050 or MED-4750 polymers were impregnated with ACV in a 1 : 1 : 1 ratio of component A, component B, and ACV, and the release of acyclovir was determined every 24 hours for 60 days. Inset shows a magnified view of days 11 to 60, after initial burst release of ACV. The rate of release of ACV from the implants was determined as described in Section 2. Briefly, implants were suspended in 1 mL of phosphate-buffered saline and removed to fresh PBS every 24 hours. Following incubation, each sample was analyzed by HPLC. ACV was quantitated based on comparison with a standard curve. Each point represents the average of five independent samples; error bars denote standard deviation of the averages.

Implants consist of a matrix of silicone and powdered ACV (Sigma-Aldrich, St. Louis, MO, and Advance Scientific & Chemical, Ft. Lauderdale, FL). Two different silicone elastomers were used, as specified in the text: either NuSil MED-4050 or NuSil MED-4750 (NuSil Silicone Technology, Carpinteria, CA; http://www.nusil.com/library/products/MED-4035_MED-4050_MED-4065P.pdf and http://www.nusil.com/library/products/MED-4750P.pdf). Each elastomer is composed of two parts, A and B. Both parts (0.20 g each) were individually softened with ten passes on a pasta maker (Laroma Manual Pasta Machine, Weston, Strongsville, OH) and then milled together for 20 passes to begin polymerization. For impregnation with ACV, drug (0.20 g to achieve a 1 : 1 : 1 ratio by weight of component A : component B : ACV as shown in Figures 1, 2, and 4; 0.04–0.20 g as specified in the text for Figure 3) was added to the mixture and milled for 20 more passes, followed by implant formation. This method was described in [37].

To create rod-shaped implants, the softened material was placed in a 0.5-inch i.d. polycarbonate cylinder, fitted with a matching piston, and extruded through a 2-mm diameter die under pressure in a one-ton arbor press (Harbor Freight Tools, Parkville, MD). The cylinder, piston, and die apparatus were built by Robert Kuta of the Fisher College of Science and Mathematics (Towson University Department of Physics and Geosciences Machine Shop). Implants were cured at room temperature for 7 days, then at 60°C for 24 hours [38]. After the curing process, implants were cut to 15-mm lengths. Implants were sterilized by soaking in 40% sodium hypochlorite (household bleach) for one minute at room temperature, transferred through four changes of PBS for 30 seconds each to remove the bleach, and then air-dried.

### 2.2. Determining the Efficacy of Implants at Preventing CPE in Cell Culture

A 24-well culture plate was prepared with 1 mL/well of Dulbecco's modified Eagle's medium (DMEM; Invitrogen, Carlsbad, CA) supplemented with 10% fetal bovine serum (Gemini Bio-Products, West Sacramento, CA) and 1% penicillin-streptomycin-amphotericin B (Mediatech Inc., Herndon, VA). For analysis of HSV-2, African green monkey kidney cells (Vero cells, ATCC CCL-81; gift of Prashant Desai, Johns Hopkins University School of Medicine) were added to each well and grown for 24 hours at 37°C and 5% CO_2_ to about 80% confluence (approx. 1.6 × 10^5^ cells/well). A single acyclovir-containing silicone implant was placed into each “implant” well. Control wells received either acyclovir in PBS (PBS/ACV) to a final concentration of 0.22 *μ*g/mL or no drug. One or two days later, wells were inoculated with 95 plaque-forming units of HSV-2 (G) (gift of Kevin Yim, Virion Systems, Inc.) or HSV-2 (MS) (ATCC #VR-540; ATCC, Manassas, VA), as noted. Digital photos were taken 3 days after infection, except where noted. Mock-infected wells received complete DMEM without virus. Acyclovir-free control wells (“None”) received neither ACV nor implant. For analysis of VZV, MRC-5 cells (ATCC #CCL-171; ATCC) were added to each well and grown for 24 hours at 37°C and 5% CO_2_ to about 80% confluence (approx. 1.6 × 10^5^ cells/well). A single acyclovir-containing silicone rod was placed into each “implant” well. Control wells received either acyclovir in PBS (PBS/ACV) to a final concentration of 10 *μ*g/mL or no drug. Two days later, wells were inoculated with 150 plaque-forming units of VZV (Oka) (ATCC #VR-795; ATCC, Manassas, VA). Digital photos were taken 10 days after infection. Mock-infected cells received complete DMEM without virus. Acyclovir-free control wells (“None”) received neither ACV nor implant. HSV-2 (MS) titers were determined by limiting dilution of a 100 *μ*L sample removed 3 days after infection [39].

### 2.3. Determining *In Vitro* Release Kinetics

The rate of release of ACV from the implants *in vitro* was determined by HPLC analysis. 

To determine ACV levels in culture, 100 *μ*L of tissue culture supernatant from infected cell cultures (above) was removed 3 days after infection. Penciclovir (1 *μ*g) was included as an internal control, to monitor efficiency of ACV recovery, and proteins were precipitated with 1 mL methanol. Precipitates were removed by centrifugation, and then supernatants were dried in a Speed-Vac apparatus, resuspended in 1 mL of 1 : 9 water : acetonitrile, and then passed through a 0.45 *μ* syringe filter (Phenex-RC 4 mm; Phenomenex, Torrance, CA) for HPLC analysis (see the following).

For longer term analysis of release, implants were placed into 1.5-mL microcentrifuge tubes with 1.0 mL of phosphate-buffered saline (HyQ PBS; HyClone, Logan, UT) at pH 7.5 and held at 25°C. Every 24 h for 60 days, each implant was moved into a new microcentrifuge tube with fresh PBS.

ACV was quantitated by high performance liquid chromatography (HPLC). Each sample (50 *μ*L) was prepared for analysis by dilution with 450 *μ*L acetonitrile and analyzed on an Agilent system equipped with a Luna HILIC 3 micron 15 × 100 mm column (Phenomenex, Torrance, CA) under isocratic conditions running 90% acetonitrile/10% formic acid (0.1%) as the mobile phase. Values were calculated against ACV standards run simultaneously.

## 3. Results

### 3.1. Efficacy of Silicone-ACV Implant against HSV-2 and VZV

Previously, we demonstrated that ACV-impregnated silicone implants exhibit antiviral activity against HSV-1 in Vero cells. We sought to determine whether the implants would show similar activity against other herpesviruses. To test activity against two strains of HSV-2, Vero cells were incubated with no drug, ACV in solution, or MED-4050 silicone implants impregnated with ACV in a 1 : 1 : 1 ratio (by weight) of component A : component B : ACV (see Section 2). Two days after adding ACV or implant to the cells, wells were inoculated with either HSV-2 (G), HSV-2 (MS), or medium alone (“Mock”). Three days after challenge, all mock-infected cells remained healthy, as did virally challenged cells that received ACV in solution or the MED-4050/ACV implant; all maintained a confluent monolayer (Figure 1(a)). However, cells infected with HSV-2 that received neither ACV in solution nor ACV-impregnated implant exhibited significant cytopathic effect (CPE), including cell rounding and the appearance of large cleared areas.

To test the efficacy of ACV-impregnated silicone implants against varicella zoster virus (VZV), MRC-5 cells were incubated with no drug, ACV in solution, or MED-4050 silicone implant impregnated with ACV. Two days later, half the wells were challenged with VZV (Oka), while the rest received medium alone (“Mock”). Ten days after infection, all mock-infected cells remained healthy, appearing as a confluent monolayer of striated fibroblasts (Figure 1(b)). Cells incubated with ACV in solution or the MED-4050/ACV implant were indistinguishable from mock-infected controls. In contrast, VZV-infected cells that did not receive any drug exhibited CPE in the form of pitting of the monolayer.

### 3.2. Comparing Two Polymers

We demonstrated previously that silicone-ACV implants generated with the MED-4050 polymer provide protection against HSV-1 recrudescence in mice. MED-4050 has a significant drawback, however, in that its use in humans is restricted to a maximum of 29 consecutive days. MED-4750 has similar physical properties to MED-4050, but it is employed in devices approved by the US Food and Drug Administration for unlimited use in humans. We therefore sought to compare the efficacy of these two polymers as delivery vehicles for ACV. When combined in a 1 : 1 : 1 ratio (by weight) of component A : component B : ACV, implants generated from both polymers protected Vero cells from an HSV-2 challenge (Figure 2(a)). Vero cells that received an ACV-impregnated implant maintained a smooth monolayer following the HSV-2 challenge, as did those that received ACV in solution. Both implant types appreciably reduced the amount of live virus produced during infection (Figure 2(b)). In contrast, cells that did not receive ACV and were infected showed significant CPE. Furthermore, the levels of ACV delivered by each implant type (Figure 2(c)) were comparable to the levels found over a longer term *in vitro* drug delivery study (Section 3.3). Therefore, the ACV-impregnated MED-4750 polymer is capable of protecting cultured cells from an HSV-2 challenge, comparable in efficacy to implants generated with the MED-4050 polymer.

### 3.3. Determination of Minimum Effective Dose of ACV in Silicone Implants

To determine the minimum effective dose of ACV *in vitro*, we generated MED-4750 silicone implants with varying ratios of polymer to ACV. Because the polymer consists of equal ratios of two components (A and B), this is expressed as a three-part ratio by weight of MED-4750 component A : MED-4750 component B : ACV. Implants were generated with decreasing amounts of ACV, in the ratios 1 : 1 : 1 (33%), 3 : 3 : 2 (25%), 2 : 2 : 1 (20%), 3 : 3 : 1 (14%), and 5 : 5 : 1 (9%). All implants provided protection against HSV-2 infection of Vero cells when observed three days after the challenge, as compared to cells that received no implant or implant without ACV (Figure 3). When cells were examined five days after the challenge, implants with the four highest concentrations of ACV still provided protection. However, CPE was observed at day 5 in HSV-2 infected cells treated with the implant containing 9% ACV (Figure 3, 9% ACV, lowest panel). In contrast, cells that received higher doses of ACV remained unchanged on day five (not shown). The lowest dose implant that remained effective at day five contained 14% ACV.

### 3.4. *In Vitro *Release Kinetics of ACV from Implants Made from MED-4050 and MED-4750 Polymers

Previously, we demonstrated that ACV was continually released from rod-shaped implants generated with MED-4050 polymer over a 63-day period with near-zero-order kinetics, as determined by spectrophotometric quantitation of ACV. We sought to extend these observations to ACV released by MED-4750 and to quantitate ACV by HPLC. Implants generated from both polymers were impregnated with ACV in a 1 : 1 : 1 ratio, and the rate of ACV release was determined over a 60-day period (Figure 4). Consistent with our previous observations, both implants released an initial burst of ACV, followed by a long period of slow release. However, while the burst period for MED-4750 lasted five days, MED-4050 implants delivered a longer-lived burst, which lasted approximately 10–20 days (Figure 4 and [37]). Following the initial burst, MED-4750 implants released ACV with near-zero-order kinetics for the remainder of the 60-day test period. We observed an average daily release of 1.3923 ± 0.5908 *μ*g ACV from MED-4750 implants over days 20–60. This is 72% lower than the 4.9165 ± 2.5450 *μ*g ACV released daily from MED-4050 implants over the same period.

## 4. Discussion

Clinical reactivation resulting in painful genital lesions is estimated to occur in 10–25% of individuals infected with HSV-2 [11, 12], and long-term, prophylactic ACV treatment dramatically reduces the frequency of clinical events [24, 26–28, 40]. Although most HSV-2 seropositive individuals are asymptomatic, more than three-quarters shed virus, and it is during periods of subclinical shedding in the source partner when most sexual transmission of HSV-2 takes place [12, 41–43]. Prophylactic ACV or VACV has been shown to reduce both viral shedding and sexual transmission of HSV-2 [23, 25, 28, 44] and does not lead to drug resistance in immunocompetent patients [28].

A recent analysis of HSV-2 kinetics determined that viral expansion is most rapid in the first 12 hours of a shedding episode and peaks by the second or third day. Study participants taking twice-daily ACV experienced a log reduction in the mean expansion rate during the first day of shedding relative to those taking a placebo, along with a 53% decrease in episode frequency [45, 46]. After the first 12 hours following initiation of the shedding episode, however, viral kinetics no longer respond to the presence of antiviral medication. These results support the clinical observation that ACV needs to be administered as early as possible during a recurrence in order to be effective [47]. Indeed, constant prophylactic ACV is more effective than acute treatment at reducing the duration and severity of disease, likely because it ensures that drug is present before and during the crucial early hours of viral expansion [48].

In the United States, approximately half a million people experience shingles each year, and as many as 50% of those who live to the age of 85 endure at least one episode in their lifetime [33]. Once shingles has been diagnosed, administration of antiviral drugs does not prevent its frequent sequel, a chronic neuropathic pain syndrome termed postherpetic neuralgia, or PHN [33, 35]. Long-term administration of antiviral drugs has not been tested as a strategy to prevent shingles or PHN in the general population. However, when prophylactic long-term oral therapy with ACV or VACV was tested in patients undergoing cancer chemotherapy or bone marrow transplantation, the occurrence of herpes zoster and, consequently, PHN was prevented [34, 36].

It has been suggested that widespread childhood VZV vaccination may be responsible for an observed increase in adult herpes zoster, due to a loss of natural boosting of adult immunity [49, 50]. The current recommendation for prevention of shingles is vaccination of healthy adults, which reduces the likelihood of developing herpes zoster by 51% and of PHN by 66% [51, 52]. Long-term administration of ACV may offer an additional tool for reducing the frequency and severity of herpes zoster and PHN in the elderly.

The use of an implantable device to deliver antiherpetics is not without precedent. Vitrasert (Bausch & Lomb), a polyvinyl alcohol-ethylene vinyl acetate polymer-copolymer impregnated with ganciclovir, can be implanted intravitreously to treat CMV retinitis in AIDS patients, although the device must be replaced every 5 to 8 months [53]. Vitrasert has seen limited clinical use and has never been deployed to treat human alpha herpesvirus infections.

In our previous study, the MED-4050 silicone implant released roughly 1 *μ*g of ACV daily, a fifth of what we observe for MED-4050 here [37]. This discrepancy most likely stems from the difference in previous methods that we used to quantitate ACV. In the earlier study, supernatants were subjected to several processing steps prior to spectrophotometric analysis, with a likely impact on sample retention and accuracy of measurement. In contrast, here supernatants were analyzed directly by HPLC, minimizing handling and associated sample loss. Furthermore, streamlining of the extrusion process during manufacture has likely improved the quality of our implants.

In addition to providing continuous drug delivery, localized implantable devices offer the possibility of reducing drug dose far below that which is commonly used systemically. There is still some uncertainty as to whether treatment should target the site of latency or reactivation, although the efficacy of topical antiviral medications favors the latter. All alpha herpesviruses are characterized by primarily localized latency and reactivation and therefore are potential targets for localized therapy. These include the etiologic agents of a diverse array of veterinary diseases, such as feline herpes conjunctivitis and bovine herpes mammillitis [54, 55]. 

Based on the measured release kinetics following the initial burst period, each MED-4750 implant could theoretically last for up to 17.5 years, but the kinetics of drug delivery and the minimum dose required to have a therapeutic effect would need to be determined in a true long-term *in vivo* study. To assay for local and systemic delivery of ACV from the device as well as efficacy *in vivo*, we are currently undertaking studies in an animal model.

## 5. Conclusions

Previously, we observed that an implantable silicone device impregnated with ACV offers protection against HSV-1 both *in vitro* and *in vivo *[37]. Here, we have extended our *in vitro* results to demonstrate protection against the two remaining human alpha herpesviruses, HSV-2 and VZV. We also obtained protection against HSV-2 using a silicone polymer (MED-4750) approved in devices that are deployed for long-term use in humans. A minimum dose of 14% ACV was required to obtain a full five days of protection against HSV-2. The MED-4750 implant released an initial burst of ACV lasting 5 days, followed by a steady daily release of approximately 1.4 *μ*g ACV with near-zero-order kinetics until the end of the 60-day test period.

As a preliminary step, this study has demonstrated that the antiherpetic ACV can be formulated into a long-term delivery vehicle that may function as a single antiviral intervention, one that could last considerably longer and be more easily managed than daily oral maintenance therapy.

A long-lasting, implantable drug delivery device has the potential to significantly impact the treatment of human alpha herpesvirus infections. Given the high frequency of subclinical shedding of HSV-2, continuous therapy has been suggested for those with frequent outbreaks as well as for persons with concurrent HIV infection [43]; continuous, long-term dosing would also be likely to reduce the frequency of transmission to susceptible sexual partners. The use of a slow-release implant eliminates concerns relating to patient compliance with a daily drug regimen. In VZV-positive individuals, delivery of ACV in an implantable device has the potential to reduce the incidence of both shingles and its debilitating sequel, postherpetic neuralgia. Long-term ACV use is not associated with significant acquisition of drug resistance in immunocompetent individuals [28].
